# Efficacy and safety of two doses of budesonide/formoterol fumarate metered dose inhaler in COPD

**DOI:** 10.1183/23120541.00187-2019

**Published:** 2020-04-27

**Authors:** Nicola A. Hanania, Alberto Papi, Antonio Anzueto, Fernando J. Martinez, Kimberly A. Rossman, Christy S. Cappelletti, Elizabeth A. Duncan, Jack S. Nyberg, Paul M. Dorinsky

**Affiliations:** 1 Section of Pulmonary and Critical Care Medicine, Baylor College of Medicine, Houston, TX, USA; 2 Research Centre on Asthma and COPD, Dept of Medical Sciences, University of Ferrara, Ferrara, Italy; 3 Pulmonary Medicine and Critical Care, University of Texas Health Science Center and South Texas Veterans Health Care System, San Antonio, TX, USA; 4 Joan and Sanford I. Weill Dept of Medicine, Weill Cornell Medicine, New York, NY, USA; 5 AstraZeneca, Morristown, NJ, USA; 6 AstraZeneca, Durham, NC, USA; 7 Former employee of AstraZeneca, Morristown, NJ, USA

## Abstract

Inhaled corticosteroid/long-acting β_2_-agonist combination therapy is a recommended treatment option for patients with chronic obstructive pulmonary disease (COPD) and increased exacerbation risk, particularly those with elevated blood eosinophil levels. SOPHOS (NCT02727660) evaluated the efficacy and safety of two doses of budesonide/formoterol fumarate dihydrate metered dose inhaler (BFF MDI) *versus* formoterol fumarate dihydrate (FF) MDI, each delivered using co-suspension delivery technology, in patients with moderate-to-very severe COPD and a history of exacerbations.

In this phase 3, randomised, double-blind, parallel-group, 12–52-week, variable length study, patients received twice-daily BFF MDI 320/10 µg or 160/10 µg, or FF MDI 10 µg. The primary endpoint was change from baseline in morning pre-dose trough forced expiratory volume in 1 s (FEV_1_) at week 12. Secondary and other endpoints included assessments of moderate/severe COPD exacerbations and safety.

The primary analysis (modified intent-to-treat) population included 1843 patients (BFF MDI 320/10 µg, n=619; BFF MDI 160/10 µg, n=617; and FF MDI, n=607). BFF MDI 320/10 µg and 160/10 µg improved morning pre-dose trough FEV_1_ at week 12 *versus* FF MDI (least squares mean differences 34 mL [p=0.0081] and 32 mL [p=0.0134], respectively), increased time to first exacerbation (hazard ratios 0.827 [p=0.0441] and 0.803 [p=0.0198], respectively) and reduced exacerbation rate (rate ratios 0.67 [p=0.0001] and 0.71 [p=0.0010], respectively). Lung function and exacerbation benefits were driven by patients with blood eosinophil counts ≥150 cells·mm^−3^. The incidence of adverse events was similar, and pneumonia rates were low (≤2.4%) across treatments.

SOPHOS demonstrated the efficacy and tolerability of BFF MDI 320/10 µg and 160/10 µg in patients with moderate-to-very severe COPD at increased risk of exacerbations.

## Abstract 2

**Co-suspension delivery technology budesonide/formoterol fumarate metered dose inhaler improve lung function and reduce exacerbation risk *versus* LABA monotherapy in patients with moderate to very severe COPD and an exacerbation history in the prior year** http://bit.ly/3aDOvru

## Introduction

The Global Initiative for Chronic Obstructive Lung Disease (GOLD) recommends the use of an inhaled corticosteroid (ICS) as an add-on treatment with a long-acting β_2_-agonist (LABA) for patients with chronic obstructive pulmonary disease (COPD) who experience exacerbations despite long-acting bronchodilator monotherapy; particularly when they have elevated blood eosinophil levels [1]. Clinical studies have demonstrated that ICS/LABA combination therapy can improve lung function and reduce COPD exacerbation rates more effectively than LABA monotherapy [2–11].

The budesonide/formoterol fumarate dihydrate metered dose inhaler (BFF MDI) is an ICS/LABA fixed-dose combination that is formulated using innovative co-suspension delivery technology. Unlike drug crystal-only suspensions, co-suspension delivery technology enables reliable aerosol performance and consistent drug delivery, even in the presence of simulated patient-handling errors [12, 13].

The recently completed phase 3 TELOS study (NCT02766608), conducted in patients with moderate-to-very severe COPD who were not required to have an exacerbation history, demonstrated that BFF MDI 320/10 µg and 160/10 µg improved lung function over 24 weeks compared with ICS monotherapy (which is not a recommended therapy for COPD) and reduced COPD exacerbations compared with LABA monotherapy. There was a numerical dose response favouring the BFF MDI 320/10 µg dose for all lung function and exacerbation endpoints [11].

The current SOPHOS study (NCT02727660) evaluated lung function (primary objective), COPD exacerbations (secondary objectives) and safety, following treatment with BFF MDI 320/10 µg, BFF MDI 160/10 µg or formoterol fumarate dihydrate (FF) MDI monotherapy for at least 12, and up to 52, weeks in patients with moderate-to-very severe COPD who had a documented history of at least one moderate/severe COPD exacerbation in the previous 12 months.

## Material and methods

### Patients

Patients (40–80 years of age) were current/former smokers (≥10 pack-years) with a history of COPD (defined by American Thoracic Society/European Respiratory Society or locally applicable guidelines) [14] who remained symptomatic (COPD Assessment Test score ≥10) despite receiving one or more inhaled maintenance bronchodilators. Patients were required to have a post-bronchodilator forced expiratory volume in 1 s (FEV_1_)/forced vital capacity ratio <0.70, and post-bronchodilator FEV_1_ ≥25% to <80% predicted normal (second screening visit) according to the National Health and Nutrition Examination Survey III reference equations [15, 16]. Patients had a documented history of at least one moderate/severe COPD exacerbation in the previous 12 months. Patients with a current diagnosis of asthma were excluded.

### Study design

In this double-blind, parallel-group study, patients were screened at 292 centres in 18 countries, and randomised 1:1:1 using an interactive web response system to receive twice-daily BFF MDI 320/10 μg, BFF MDI 160/10 μg or FF MDI 10 μg (figure E1). Randomisation was stratified by country, exacerbation history, post-bronchodilator FEV_1_ and blood eosinophil count (with enrolment targeted at a 2:1 ratio of patients with eosinophil counts ≥150 and <150 cells·mm^−3^).

Prohibited COPD medications, which included long-acting muscarinic antagonists (LAMAs) and LABAs (see online data supplement), were discontinued at the start of screening. Patients receiving maintenance ICS (single inhaler or ICS/LABA) continued the ICS component of treatment throughout the screening period and discontinued the ICS component at randomisation. Albuterol sulfate (Ventolin HFA; GlaxoSmithKline, Research Triangle Park, NC, USA) was provided as rescue medication throughout the study.

Patients received 12 to 52 weeks of treatment. The study ended when the last patient remaining in the study completed 12 weeks of treatment or completed their final visit.

The study adhered to the Declaration of Helsinki, the International Council for Harmonisation/Good Clinical Practice and applicable regulatory requirements. Patients provided written informed consent. Informed consent forms were reviewed and approved by an Independent Ethics Committee or Institutional Review Board.

### Efficacy endpoints and assessments

Efficacy analyses were based on the modified intent-to-treat (mITT) population (data from all patients who received any amount of the study drug collected before treatment discontinuation). Endpoints and statistical analysis approaches differed depending on regional regulatory requirements. This manuscript describes the analysis using the US approach. Further details of study estimands, analysis populations, statistical analyses, sample size calculations, definitions of COPD exacerbations and severity, the analysis using the Ex-US approach, and *post hoc* and subgroup analyses are provided in the online data supplement.

The primary endpoint (US registration approach) was the change from baseline in morning pre-dose trough FEV_1_ at week 12, analysed using the efficacy estimand. Analysis of the same outcome using an attributable estimand was the first secondary endpoint.

Further secondary endpoints were: time to first moderate/severe COPD exacerbation; change from baseline in average daily rescue medication use over 12 weeks; and St George's Respiratory Questionnaire (SGRQ) responder rate (percentage of patients achieving a minimal clinically important difference ≥4 units in total score) at week 12. The rates of moderate/severe exacerbations were also assessed.

Pre-specified subgroup analyses of the primary endpoint and exacerbation rates were performed according to baseline blood eosinophil count (<150 *versus* ≥150 cells·mm^−3^) and according to number of moderate/severe COPD exacerbations in the previous 12 months (1 *versus* ≥2). Additional *post hoc* analyses of exacerbation rates by ICS use at baseline and by 4-week intervals were also performed.

### Safety analyses

Treatment-emergent adverse events (TEAEs) were monitored in the safety population (all randomised patients who received study drug, even if <1 full dose). Potential major adverse cardiovascular events (MACE) and pneumonia cases were adjudicated by an external Clinical Endpoint Committee.

## Results

### Study population

**FIGURE 1:**
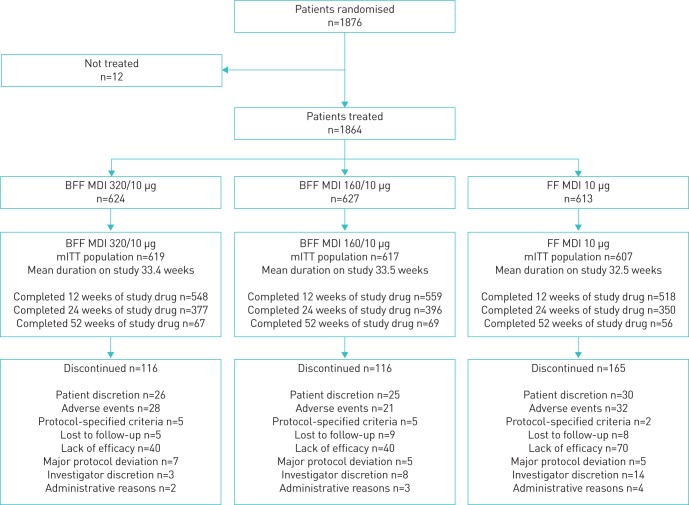
Patient disposition. A total of 21 subjects had participated in multiple sponsor studies and were excluded from the intent to treat (ITT), modified ITT (mITT) and safety populations. BFF: budesonide/formoterol fumarate dihydrate; MDI: metered dose inhaler; FF: formoterol fumarate dihydrate.

The first patient was randomised in May 2016, and the study completed in April 2018. Of 1876 randomised patients, 1864 (99.4%) were treated (BFF MDI 320/10 µg, n=624; BFF MDI 160/10 µg, n=627; and FF MDI, n=613), and 1843 (98.2%) were included in both the mITT and safety populations; A total of 1585 (84.5%) patients completed the study (fig. 1). Overall, 1625 (86.6%), 1123 (59.9%) and 192 (10.2%) patients completed 12, 24 and 52 weeks of treatment, respectively. The mean study duration was 232.1 days (33.2 weeks). Reasons for study discontinuation are summarised in figure 1.

Demographic characteristics were generally balanced across treatment groups (table 1). Most patients in the mITT population were white (83.2%) and male (57.0%). The mean±standard deviation age was 64.9±8.3 years, 39.3% of patients were current smokers and the mean±sd smoking history was 45.0±27.1 pack-years. Most patients (76.3%) used ICS at screening. Enrolment had been targeted to achieve a 2:1 ratio for eosinophil subgroups (≥150 *versus* <150 cells·mm^−3^) and, accordingly, 67.2% of patients had a baseline blood eosinophil count of ≥150 cells·mm^−3^ (figure E2). In the 12 months before screening, 38.5% of patients had two or more moderate/severe exacerbations.

**TABLE 1:** Demographics and baseline characteristics (modified intent-to-treat population)

	**BFF MDI 320/10 µg**	**BFF MDI 160/10 µg**	**FF MDI 10 µg**	**All patients**
**Patients, n**	619	617	607	1843
**Age, years**	65.3±8.1	64.5±8.4	64.8±8.5	64.9±8.3
**Age ≥65 years**	347 (56.1)	318 (51.5)	334 (55.0)	999 (54.2)
**Male**	367 (59.3)	345 (55.9)	339 (55.8)	1051 (57.0)
**White/black/other, %**	83.0/4.8/12.1	83.6/3.6/12.8	83.0/4.8/12.2	83.2/4.4/12.4
**Current smoker**	257 (41.5)	223 (36.1)	245 (40.4)	725 (39.3)
**Number of pack-years smoked**	44.2±26.0	45.8±28.0	44.9±27.4	45.0±27.1
**Total CAT score^#^**	21.7±6.4	21.3±6.1	21.2±6.3	21.4±6.3
**SGRQ total score**	51.4±18.2	51.1±17.8	50.7±17.6	51.1±17.8
**History of moderate/severe exacerbations**				
0 exacerbations	5 (0.8)	2 (0.3)	1 (0.2)	8 (0.4)
1 exacerbation	378 (61.1)	374 (60.6)	374 (61.6)	1126 (61.1)
≥2 exacerbations	236 (38.1)	241 (39.1)	232 (38.2)	709 (38.5)
**History of severe exacerbations**				
0 exacerbations	494 (79.8)	510 (82.7)	497 (81.9)	1501 (81.4)
1 exacerbation	108 (17.4)	96 (15.6)	93 (15.3)	297 (16.1)
≥2 exacerbations	17 (2.7)	11 (1.8)	17 (2.8)	45 (2.4)
**COPD severity**				
Mild	0 (0.0)	1 (0.2)	1 (0.2)	2 (0.1)
Moderate	338 (54.6)	330 (53.5)	321 (52.9)	989 (53.7)
Severe	241 (38.9)	230 (37.3)	244 (40.2)	715 (38.8)
Very severe	37 (6.0)	56 (9.1)	36 (5.9)	129 (7.0)
**COPD duration, years**	8.1±6.4	7.7±5.5	7.9±6.2	7.9±6.1
**Prior COPD treatment^¶^**				
SABA and/or SAMA only, PRN	4 (0.6)	3 (0.5)	1 (0.2)	8 (0.4)
MA only	26 (4.2)	33 (5.3)	30 (4.9)	89 (4.8)
BA only	22 (3.6)	37 (6.0)	39 (6.4)	98 (5.3)
ICS only	12 (1.9)	14 (2.3)	13 (2.1)	39 (2.1)
MA/BA only	83 (13.4)	81 (13.1)	71 (11.7)	235 (12.8)
ICS/BA only	272 (43.9)	264 (42.8)	258 (42.5)	794 (43.1)
ICS/MA only	13 (2.1)	16 (2.6)	17 (2.8)	46 (2.5)
ICS/MA/BA	185 (29.9)	165 (26.7)	177 (29.2)	527 (28.6)
**ICS use at screening**	482 (77.9)	459 (74.4)	465 (76.6)	1406 (76.3)
**Baseline blood eosinophil count**				
<150 cells·mm^−3^	197 (31.8)	213 (34.5)	193 (31.8)	603 (32.7)
≥150 cells·mm^−3^	422 (68.2)	403 (65.3)	413 (68.0)	1238 (67.2)
**Post-bronchodilator FEV_1_, % predicted**	51.09±13.70	50.59±14.27	50.97±13.46	50.88±13.81
**Post-bronchodilator reversibility for FEV_1_, %^+^**	12.1±13.0	13.2±14.1	12.9±14.3	12.7±13.8
**Rescue medication use, puffs·day^-1^**	3.5±3.2	3.7±3.4	3.6±3.5	3.6±3.4

Data are presented as mean±standard deviation or n (%), unless otherwise stated. BFF: budesonide/formoterol fumarate dihydrate; MDI: metered dose inhaler; FF: formoterol fumarate dihydrate; CAT: Chronic Obstructive Pulmonary Disease (COPD) Assessment Test; SGRQ: St George's Respiratory Questionnaire; SABA: short-acting β_2_-agonist; SAMA: short-acting muscarinic antagonist; PRN: as needed; MA: muscarinic antagonist; BA: β_2_-agonist; ICS: inhaled corticosteroid; FEV_1_: forced expiratory volume in 1 s. ^#^: total CAT score was the sum of eight CAT item scores and could range from 0 to 40; ^¶^: scheduled use of a SAMA and/or SABA was included in all categories except PRN; ^+^: reversibility is defined as (change from pre-bronchodilator to post-bronchodilator for FEV_1_)×100/pre-bronchodilator FEV_1_.

### Efficacy analyses

**FIGURE 2:**
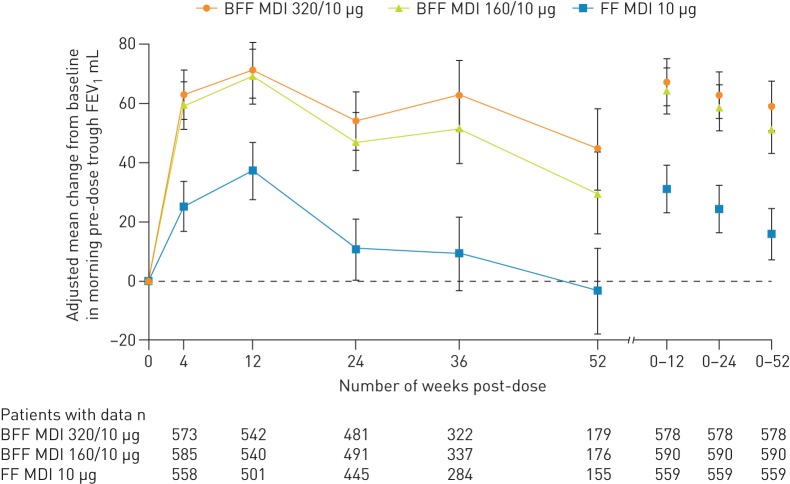
Primary efficacy endpoint: adjusted mean change from baseline in morning pre-dose trough forced expiratory volume in 1 s (FEV_1_) over time (modified intent-to-treat population; efficacy estimand). Error bars represent the standard error. BFF: budesonide/formoterol fumarate dihydrate; MDI: metered dose inhaler; FF: formoterol fumarate dihydrate.

For the primary endpoint of change from baseline in morning pre-dose trough FEV_1_ at week 12, analysed using the efficacy estimand, BFF MDI 320/10 µg and 160/10 µg demonstrated statistically significant improvements *versus* FF MDI, with least squares mean (LSM) differences of 34 mL (95% confidence interval [CI] 9–60; p=0.0081) and 32 mL [95% CI 7–57; p=0.0134], respectively (table 2 and fig. 2). Analysis of the same endpoint for the attributable estimand (secondary analysis) also demonstrated significant improvements for BFF MDI 320/10 µg and 160/10 µg *versus* FF MDI (LSM differences 50 mL [95% CI 24–76, p=0.0002] and 44 mL [95% CI 18–70; p=0.0009], respectively) (table 2).

**TABLE 2:** Primary, secondary and additional efficacy endpoints (modified intent-to-treat population; efficacy estimand unless stated otherwise)

	**BFF MDI 320/10 μg**	**BFF MDI 160/10μg**	**FF MDI 10 µg**
**Patients, n**	619	617	607
**Primary endpoint**			
Change from baseline in morning pre-dose trough FEV_1_ (mL) at week 12^#^			
n	542	540	501
LSM±se	72±9.4	69±9.3	37±9.6
LSM difference *versus* FF MDI (95% CI)	34 (9–60)	32 (7–57)	
p*-*value	0.0081	0.0134	
**Secondary endpoints**			
Change from baseline in morning pre-dose trough FEV_1_ (mL) at week 12 (attributable estimand)^#^			
n	612	612	601
LSM±se	59±9.7	53±9.5	9±10.1
LSM difference *versus* FF MDI (95% CI)	50 (24–76)	44 (18–70)	
p-value	0.0002	0.0009	
Time to first moderate/severe COPD exacerbation^¶^			
Patients with exacerbation n (%)	220 (35.5)	223 (36.1)	241 (39.7)
HR *versus* FF MDI (95% CI)	0.827 (0.688–0.995)	0.803 (0.668–0.966)	
p-value	0.0441	0.0198	
SGRQ responder rate at week 12^+^			
Responders n (%)	315 (51.98)	331 (54.00)	262 (43.96)
Difference *versus* FF MDI % (95% CI)	7.55 (1.67–13.43)	11.05 (5.18–16.91)	
p-value	0.0121	0.0002	
Change from baseline in mean daily rescue medication use (puffs·day^-1^) over 12 weeks^#^			
n	612	610	599
LSM±se	−0.9±0.09	−0.9±0.09	−0.6±0.09
LSM difference *versus* FF MDI (95% CI)	−0.32 (−0.56 to −0.08)	−0.29 (−0.53 to −0.05)	
p-value	0.0097	0.0185	
**Additional endpoints**			
Rate of moderate/severe exacerbations per year^§^			
Adjusted rate of exacerbations+/−SE	0.93±0.073	0.98±0.076	1.39±0.106
Rate ratio *versus* FF MDI (95% CI)	0.67 (0.54–0.82)	0.71 (0.58–0.87)	
p*-*value	0.0001^ƒ^	0.0010^ƒ^	
Time to first moderate/severe COPD exacerbation in patients with a history of ≥2 COPD exacerbations in the last 12 months^§^			
Patients with exacerbation, n (%)	93 (39.4)	97 (40.2)	106 (45.7)
HR *versus* FF MDI (95% CI)	0.783 (0.590–1.039)	0.799 (0.603–1.059)	
p-value	0.0904	0.1178	

BFF: budesonide/formoterol fumarate dihydrate; MDI: metered dose inhaler; FF: formoterol fumarate dihydrate; FEV_1_: forced expiratory volume in 1 s; LSM: least squares mean; HR: hazard ratio; SGRQ: St George's Respiratory Questionnaire; COPD: chronic obstructive pulmonary disease. ^#^: analysed by repeated measures linear mixed model; ^¶^: analysed by Cox regression; ^+^: analysed by logistic regression model; ^§^: analysed by negative binomial regression. Rate of exacerbations per year=total number of exacerbations / total years of exposure across all patients for the treatment. ^ƒ^: p-values for this endpoint are considered nominal since these analyses were not under Type I error control.

**FIGURE 3:**
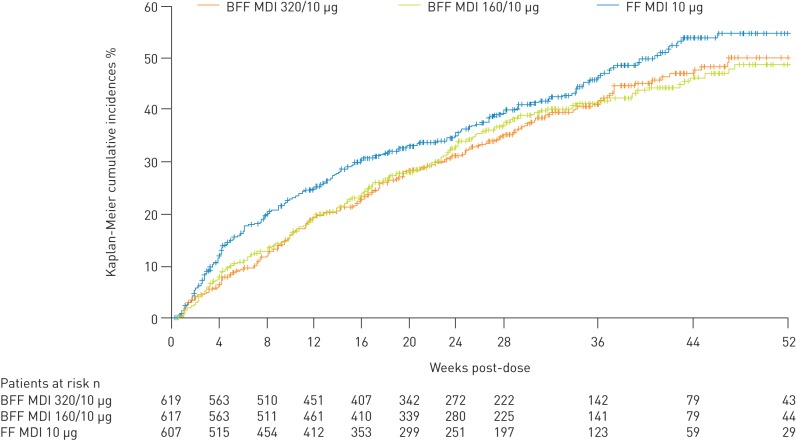
Time to first moderate/severe chronic obstructive pulmonary disease (COPD) exacerbation (modified intent-to-treat population; efficacy estimand). BFF: budesonide/formoterol fumarate dihydrate; MDI: metered dose inhaler; FF: formoterol fumarate dihydrate.

The time to first moderate/severe COPD exacerbation was significantly longer for BFF MDI 320/10 µg and BFF MDI 160/10 µg *versus* FF MDI (hazard ratio [HR] 0.827 [95% CI 0.688–0.995; p=0.0441] and 0.803 [95% CI 0.668–0.966; p=0.0198], respectively) (table 2), with the benefits for both BFF MDI doses *versus* FF MDI evident from as early as 4 weeks and throughout the treatment period (fig. 3). In the subgroup of patients with a history of at least two moderate/severe exacerbations in the last 12 months, BFF MDI 320/10 µg (n=236) and 160/10 µg (n=241) delayed the time to first exacerbation *versus* FF MDI (n=232) to a similar extent compared with the overall population. However, treatment differences did not reach statistical significance in this smaller patient sample (HR 0.783 [95% CI 0.590–1.039; p=0.0904] and 0.799 [95% CI 0.603–1.059; p=0.1178], respectively).

BFF MDI 320/10 µg and 160/10 µg reduced the rate of moderate/severe COPD exacerbations *versus* FF MDI (rate ratios 0.67 [95% CI 0.54–0.82; p=0.0001] and 0.71 [95% CI 0.58–0.87; p=0.0010], respectively; these endpoints were not included in the Type I error control strategy, so were nominally significant) (table 2).

Both BFF MDI doses significantly reduced mean daily rescue medication use over 12 weeks and significantly increased SGRQ responder rates at week 12 *versus* treatment with FF MDI alone (table 2).

**FIGURE 4:**
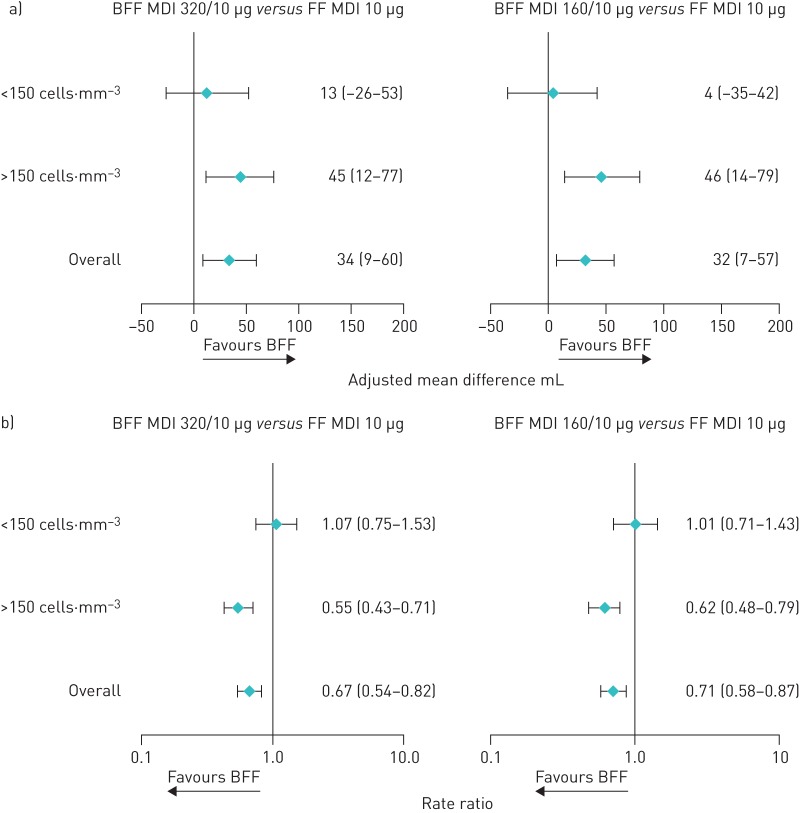
Change from baseline in morning pre-dose trough forced expiratory volume in 1 s (FEV_1_) at week 12, a) and rate of moderate/severe chronic obstructive pulmonary disease (COPD) exacerbations, b) by baseline eosinophil count (modified intent-to-treat population; efficacy estimand). Error bars represent 95% confidence intervals. BFF: budesonide/formoterol fumarate dihydrate; MDI: metered dose inhaler; FF: formoterol fumarate dihydrate.

**FIGURE 5:**
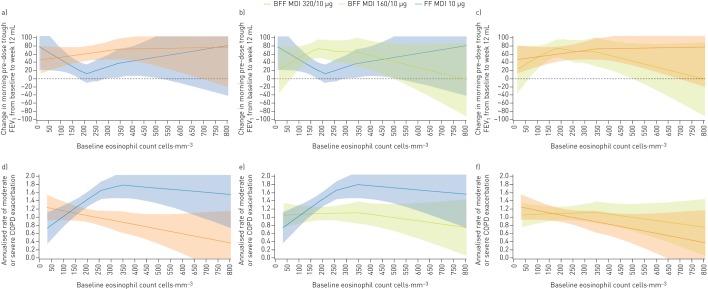
Lung function and exacerbation responses by blood eosinophil counts (efficacy estimand, modified intent-to-treat population). Change from baseline in morning pre-dose trough forced expiratory volume in 1 s (FEV_1_) (mL) at week 12 by blood eosinophil count, for a) budesonide/formoterol fumarate dihydrate (BFF) metered dose inhaler (MDI) 320/10 µg *versus* formoterol fumarate dihydrate (FF) MDI 10 µg, b) BFF MDI 160/10 µg *versus* FF MDI 10 µg, and c) BFF MDI 320/10 µg *versus* BFF MDI 160/10 µg, and rate of moderate/severe COPD exacerbations by blood eosinophil count for d) BFF MDI 320/10 µg *versus* FF MDI 10 µg, e) BFF MDI 160/10 µg *versus* FF MDI 10 µg, and f) BFF MDI 320/10 µg *versus* BFF MDI 160/10 µg. The banded areas represent 95% confidence intervals. COPD: chronic obstructive pulmonary disease.

Pre-specified subgroup analyses suggested that the improvements in morning pre-dose trough FEV_1_ at week 12 and reductions in the rate of moderate/severe COPD exacerbations with both doses of BFF MDI compared with FF MDI were driven by patients with a baseline blood eosinophil count ≥150 cells·mm^−3^ (fig. 4). Further analysis of lung function response by continuous baseline eosinophil count with locally weighted scatterplot smoothing (LOESS) curves showed that improvements in morning pre-dose trough FEV_1_ for BFF MDI 320/10 µg *versus* FF MDI at week 12 began at blood eosinophil levels of approximately 100–150 cells·mm^−3^. Results for BFF MDI 160/10 µg *versus* FF MDI were consistent with these findings up to eosinophil counts of approximately 350 cells·mm^−3^, after which improvements to lung function with BFF MDI 160/10 µg began to decrease (fig. 5a and b). LOESS curves of BFF MDI 320/10 µg and BFF MDI 160/10 µg showed that the two doses began to diverge from each other at blood eosinophil levels of approximately 350–400 cells·mm^−3^, with the treatment difference between the two doses exceeding 20 mL from approximately 450 cells·mm^−3^ (fig. 5c). Analysis of the rate of moderate/severe exacerbations by continuous baseline eosinophil count showed numerical improvements with both doses of BFF MDI *versus* FF MDI began at blood eosinophil levels of approximately 100–150 cells·mm^−3^ and were maintained with increasing eosinophil levels (fig. 5d and e). With both BFF MDI doses, improvements in the rate of moderate or severe COPD exacerbations increased with increasing blood eosinophil levels. When plotted together, the BFF MDI doses began to diverge from each other at blood eosinophil levels of approximately 150–200 cells·mm^−3^, with a treatment difference between the two doses exceeding 15% at blood eosinophil levels above approximately 250–300 cells·mm^−3^ (fig. 5f).

Subgroup analyses based on the number of moderate/severe exacerbations in the year before the study showed that improvements in lung function and exacerbation rates were similar in patients with a history of one moderate/severe exacerbation and those with at least two moderate/severe exacerbations (figure E3).

*Post hoc* analyses of the rate of moderate/severe exacerbations by ICS use prior to study entry demonstrated the benefit of ICS in all patients regardless of ICS usage prior to study entry. In an analysis that estimated the rate of exacerbations for each 4-week period, both BFF MDI doses separated from FF MDI during the first 4 months and the last 5 months of the treatment period, with some overlap during the mid-portion of the treatment period. Further details are provided in the online data supplement.

### Safety

The incidence of TEAEs (table 3) was similar across treatment groups (BFF MDI 320/10 µg, 48.0%; BFF MDI 160/10 µg, 50.9%; FF MDI, 50.2%). Most TEAEs were mild or moderate, and few patients (4.4%) experienced a TEAE that was considered to be drug related. The incidence of serious TEAEs was highest for FF MDI (13.7%), followed by BFF MDI 160/10 µg (12.6%) and BFF MDI 320/10 µg (9.7%); few serious TEAEs (0.2%) were considered to be drug related. The incidences of TEAEs and serious TEAEs leading to study drug discontinuation were similar across the treatment groups.

**TABLE 3:** Treatment-emergent adverse events (TEAEs) (safety population)

	**BFF MDI 320/10 μg**	**BFF MDI 160/10 μg**	**FF MDI 10 μg**	**All patients**
**Patients, n**	619	617	607	1843
**≥1 TEAE**	297 (48.0), 819	314 (50.9), 919	305 (50.2), 932	916 (49.7), 2670
Mild	124 (20.0)	116 (18.8)	106 (17.5)	346 (18.8)
Moderate	118 (19.1)	135 (21.9)	119 (19.6)	372 (20.2)
Severe	55 (8.9)	63 (10.2)	80 (13.2)	198 (10.7)
**Drug-related TEAEs^#^**	31 (5.0), 43	26 (4.2), 35	24 (4.0), 33	81 (4.4), 111
**Serious TEAEs^¶^**	60 (9.7), 88	78 (12.6), 110	83 (13.7), 151	221 (12.0), 349
**Drug-related serious TEAEs**	1 (0.2), 2	1 (0.2), 1	1 (0.2), 1	3 (0.2), 4
**TEAEs leading to early discontinuation**	28 (4.5), 35	21 (3.4), 29	33 (5.4), 42	82 (4.4), 106
**Serious TEAEs leading to early discontinuation**	17 (2.7), 18	16 (2.6), 17	22 (3.6), 25	55 (3.0), 60
**Deaths (all causes)**				
On-treatment	3 (0.5)	4 (0.6)	8 (1.3)	15 (0.8)
Post-treatment	3 (0.5)	4 (0.6)	0 (0.0)	7 (0.4)
**TEAEs occurring in ≥4% of patients in any treatment arm (preferred term)**				
Nasopharyngitis	41 (6.6), 53	53 (8.6), 61	36 (5.9), 43	130 (7.1), 157
COPD	27 (4.4), 32	30 (4.9), 33	50 (8.2), 65	107 (5.8), 130
Upper respiratory tract infection	28 (4.5), 29	14 (2.3), 18	24 (4.0), 28	66 (3.6), 75
**Confirmed MACE as determined by CEC**	0 (0.0), 0	3 (0.5), 3	6 (1.0), 6	9 (0.5), 9
**Confirmed pneumonia as determined by CEC**	10 (1.6), 11	15 (2.4), 15	14 (2.3), 15	39 (2.1), 41
Serious confirmed pneumonia events	8 (1.3), 8	11 (1.8), 11	8 (1.3), 9	27 (1.5), 28

Data are presented as n (%), events or n (%), unless otherwise stated. BFF: budesonide/formoterol fumarate dihydrate; MDI: metered dose inhaler; FF: formoterol fumarate dihydrate; COPD: chronic obstructive pulmonary disease; MACE: major adverse cardiovascular event; CEC: Clinical Endpoint Committee. ^#^: relationship determined by investigator assessment; ^¶^: TEAE leading to death, hospitalisation or a life-threatening TEAE.

There were 22 deaths in the study (15 on-treatment, *i.e.* due to adverse events that started during the treatment period, and seven post-treatment). The highest number of on-treatment deaths occurred in the FF MDI group (n=8), followed by BFF MDI 160/10 µg (n=4) and BFF MDI 320/10 µg (n=3). No deaths were considered to be drug related. Details of the causes of death are provided in the online data supplement.

The incidence of confirmed pneumonia was 1.6% in the BFF MDI 320/10 μg group (n*=*10), 2.4% in the BFF MDI 160/10 µg group (n=15) and 2.3% in the FF MDI group (n=14). The proportion of patients with confirmed pneumonia was lowest in the BFF MDI 320/10 µg group over all time periods (12, 24, 36 and 52 weeks; table E1). Overall, 27 patients (1.5%) had confirmed pneumonia that was considered a serious TEAE.

Nine patients experienced TEAEs (BFF MDI 160/10 µg, n=3 [0.5%]; FF MDI, n=6 [1.0%]) that were confirmed as MACE (none in the BFF MDI 320/10 µg group).

## Discussion

The SOPHOS study aimed to evaluate the efficacy and safety of BFF MDI 320/10 µg and 160/10 µg in symptomatic patients with moderate-to-very severe COPD who had a prior history of COPD exacerbations. Both doses of BFF MDI resulted in statistically significant improvements in lung function (primary efficacy endpoint) compared with FF MDI. Also, both doses of BFF MDI resulted in significant improvements in all secondary efficacy endpoints, including time to first moderate/severe exacerbation, and both doses reduced the rate of moderate/severe exacerbations, *versus* FF MDI. The small numerical dose response favouring BFF MDI 320/10 µg for improvements to lung function (trough FEV_1_) and for reducing exacerbation rates in the overall study population appeared to be more pronounced as blood eosinophil levels increased, with the doses diverging above approximately 350–400 cells and 150–200 cells for trough FEV_1_ and exacerbation rates, respectively. Results for the Ex-US analysis approach that are provided in the online data supplement were similar to the results for the US analysis approach that are presented in this manuscript.

Our findings were well aligned with the results of TELOS (NCT02766608), a 24-week study investigating the therapeutic effects of BFF MDI 320/10 µg and 160/10 µg relative to treatment with monocomponents in patients with moderate-to-very severe COPD [11]. In TELOS, both doses of BFF MDI also showed superiority to FF MDI monotherapy, with a dose-related response for lung function and exacerbation endpoints. However, while >70% of patients in TELOS did not have a moderate/severe COPD exacerbation in the year before the study, SOPHOS specifically enrolled patients with a documented history of moderate/severe exacerbations in the year prior to the study. As expected given the differences in the patient population, the exacerbation rates in SOPHOS were considerably higher than those in TELOS, but the treatment effect of BFF MDI compared with FF MDI, as assessed by the rate ratio for COPD exacerbations, was very similar in both studies. For patients who continued treatment beyond 24 weeks and up to 52 weeks, the effects of BFF MDI on exacerbation risk seen in the first 24 weeks of treatment were sustained over the entire treatment period. Hence, SOPHOS confirmed the efficacy of BFF MDI in a population known to be at risk of exacerbations (*i.e.* in a population for which ICS/LABA is a recommended treatment option to prevent further exacerbations [1]) over a treatment period of 12 to 52 weeks.

A pre-defined subgroup analysis according to baseline blood eosinophil count showed that improvements in lung function and reductions in exacerbation rates were driven by patients with baseline blood eosinophil counts ≥150 cells·mm^−3^. These findings were generally comparable to the TELOS study [11], which included a similar eosinophil subgroup analysis. The relationship between blood eosinophil levels and response to ICS-containing therapies, with regards to reducing exacerbation risk, has also been described in other recent studies [17–21], suggesting that patients with higher eosinophil counts may experience greater therapeutic benefits from ICS than patients with lower eosinophil counts. Our emerging understanding of how eosinophils affect responses to ICS treatment is now also being reflected in the most recent GOLD report [1]. This recommends considering combination therapy with an ICS/LABA for patients who experience one exacerbation per year despite long-acting bronchodilator treatment if they have blood eosinophil levels ≥300 cells·mm^−3^, and for patients who experience two or more moderate COPD exacerbations per year (or at least one severe COPD exacerbation leading to hospitalisation) if they have eosinophil levels ≥100 cells·mm^−3^. In our study, in which 61.1% of patients had one moderate/severe exacerbation in the year prior to the study (and the other 39.9% of patients had two or more), enrichment for patients with baseline blood eosinophils ≥150 cells·mm^−3^ allowed for a detailed analysis of lung function and exacerbations by continuous eosinophil counts. This analysis showed that with both doses, benefits on lung function and exacerbations for BFF MDI *versus* FF MDI were evident over a broad range of blood eosinophil levels, with numerical improvements beginning at approximately 100–150 cells·mm^−3^. In addition, our analysis by continuous baseline eosinophil count showed that a dose response favouring BFF MDI 320/10 µg was evident from blood eosinophil levels of approximately 450 cells·mm^−3^ for lung function endpoints and approximately 250 cells·mm^−3^ for exacerbation rates. These data suggest that there is the potential for patients with COPD to derive benefit from more than one ICS dose and that overall ICS dose response is influenced appreciably by eosinophil levels. These hypotheses will be prospectively evaluated in the recently completed ETHOS study [22]. Finally, subgroup analyses based on the number of moderate/severe COPD exacerbations (1 or ≥2) in the prior year showed that the benefits on lung function and exacerbation rates for BFF MDI *versus* FF MDI were not affected by the number of exacerbations in the year before the study.

To minimise the potential for a run-in bias [23] patients in our study who were using ICS prior to the study entry continued the ICS during the screening period until randomisation. Additional analysis of exacerbation rates for each 4-week period of treatment indicated that the benefit of BFF MDI on exacerbations was not driven solely by acute ICS withdrawal in the first month after randomisation, as suggested for recent trials comparing triple therapy with LAMA/LABA dual therapies [24].

BFF MDI 320/10 μg and 160/10 μg were well tolerated in this 12–52 week study, with no new or unexpected safety findings or dose-related effects on the pattern or frequency of TEAEs, which confirmed the findings from the 24-week TELOS study [11]. ICS use in patients with COPD has been associated with an increased risk of pneumonia [1, 25, 26]. However, there is evidence to suggest that the pneumonia risk with treatments containing budesonide may be lower compared with other ICS components [27–29], and a recent pooled analysis found no significant increase in the risk of pneumonia when comparing budesonide-containing with non-budesonide-containing treatments [30]. In the present study, the percentage of patients with confirmed pneumonia events was 2.1% overall, and lowest in the BFF MDI 320/10 µg group (1.6%); this was similar to the results of TELOS and also the KRONOS study, neither of which showed a higher incidence of pneumonia in their budesonide-containing treatment arms relative to non-budesonide-containing treatment arms [11, 31]. As in the TELOS study, no ICS dose-related effect on the incidence of pneumonia was observed in this present study [11].

A potential limitation of SOPHOS was the variable length study design and the correspondingly small proportion of patients (10.2%) who completed 52 weeks of treatment. However, the effects of BFF MDI on exacerbation risk observed in the first 24 weeks of treatment were sustained after 24 weeks, and were comparable to those observed with a different formulation of BFF *versus* FF over 6 months [4]. Since only a small portion of patients completed 12 months of treatment, the study may not be optimal for assessing risk of exacerbations over the long term. However, patients in this study were recruited over an 18-month period and all seasons were well represented, reducing the potential impact of seasonal variation on exacerbation rates.

In conclusion, the SOPHOS study extended the findings from the TELOS study by demonstrating the efficacy and safety of BFF MDI, formulated using co-suspension delivery technology, in the key target population for ICS/LABA treatment; *i.e.* patients with a history of COPD exacerbations, over a treatment period of 12 to 52 weeks. BFF MDI 320/10 µg and 160/10 µg both improved lung function and reduced exacerbation risk compared to FF MDI monotherapy, with a dose response favouring the BFF MDI 320/9.6 µg dose at elevated blood eosinophil levels. Both doses of BFF MDI were well tolerated, and there was no ICS dose-related effect on safety outcomes, including the incidence of confirmed pneumonia events. The study findings confirm that BFF MDI was an effective treatment for the management of COPD in patients who had a prior exacerbation history.

## Supplementary material

10.1183/23120541.00187-2019.Supp1 **Please note:** supplementary material is not edited by the Editorial Office, and is uploaded as it has been supplied by the author.
Online data supplement Hanania_PT009003_SOPHOS_manuscript_RESUBMITTED_SUPPPL_INFO_23Mar20

Hanania_PT009003_SOPHOS_manuscript_RESUBMITTED_SUPPPL_INFO_23Mar20.docx

## References

[1] Global Initiative for Chronic Obstructive Lung Disease *2019 Report: Global Strategy for the Diagnosis, Management and Prevention of COPD*. https://goldcopd.org. Date last accessed: 28 October 2019. 10.1016/j.arbres.2019.06.001 31320191

[2] Anzueto A, Ferguson GT, Feldman G, et al. Effect of fluticasone propionate/salmeterol (250/50) on COPD exacerbations and impact on patient outcomes. COPD 2009; 6: 320–329. doi:10.1080/15412550903140881 19863361

[3] Calverley P, Pauwels R, Vestbo J, et al. Combined salmeterol and fluticasone in the treatment of chronic obstructive pulmonary disease: a randomised controlled trial. Lancet 2003; 361: 449–456. doi:10.1016/S0140-6736(03)12459-2 12583942

[4] Ferguson GT, Tashkin DP, Skärby T, et al. Effect of budesonide/formoterol pressurized metered-dose inhaler on exacerbations versus formoterol in chronic obstructive pulmonary disease: The 6-month, randomized RISE (Revealing the Impact of Symbicort in reducing Exacerbations in COPD) study. Respir Med 2017; 132: 31–41. doi:10.1016/j.rmed.2017.09.002 29229103

[5] Sharafkhaneh A, Southard JG, Goldman M, et al. Effect of budesonide/formoterol pMDI on COPD exacerbations: a double-blind, randomized study. Respir Med 2012; 106: 257–268. doi:10.1016/j.rmed.2011.07.020 22033040

[6] Tashkin DP, Rennard SI, Martin P, et al. Efficacy and safety of budesonide and formoterol in one pressurized metered-dose inhaler in patients with moderate to very severe chronic obstructive pulmonary disease: results of a 6-month randomized clinical trial. Drugs 2008; 68: 1975–2000. doi:10.2165/00003495-200868140-00004 18778120

[7] Rennard SI, Tashkin DP, McElhattan J, et al. Efficacy and tolerability of budesonide/formoterol in one hydrofluoroalkane pressurized metered-dose inhaler in patients with chronic obstructive pulmonary disease: results from a 1-year randomized controlled clinical trial. Drugs 2009; 69: 549–565. doi:10.2165/00003495-200969050-00004 19368417 PMC3580134

[8] Calverley P, Boonsawat W, Cseke Z, et al. Maintenance therapy with budesonide and formoterol in chronic obstructive pulmonary disease. Eur Respir J 2003; 22: 912–919. doi:10.1183/09031936.03.00027003 14680078

[9] Fukuchi Y, Samoro R, Fassakhov R, et al. Budesonide/formoterol *via* Turbuhaler® *versus* formoterol *via* Turbuhaler® in patients with moderate to severe chronic obstructive pulmonary disease: phase III multinational study results. Respirology 2013; 18: 866–873. 10.1111/resp.12090. 23551359

[10] Szafranski W, Cukier A, Ramirez A, et al. Efficacy and safety of budesonide/formoterol in the management of chronic obstructive pulmonary disease. Eur Respir J 2003; 21: 74–81. doi:10.1183/09031936.03.00031402 12570112

[11] Ferguson GT, Papi A, Anzueto A, et al. Budesonide/formoterol MDI with co-suspension delivery technology in COPD: the TELOS study. Eur Respir J 2018; 52: 1801334. doi:10.1183/13993003.01334-2018 30220648 PMC6383599

[12] Doty A, Schroeder J, Vang K, et al. Drug delivery from an innovative LAMA/LABA co-suspension delivery technology fixed-dose combination MDI: evidence of consistency, robustness, and reliability. AAPS PharmSciTech 2018; 19: 837–844. doi:10.1208/s12249-017-0891-1 29019170

[13] Vehring R, Lechuga-Ballesteros D, Joshi V, et al. Cosuspensions of microcrystals and engineered microparticles for uniform and efficient delivery of respiratory therapeutics from pressurized metered dose inhalers. Langmuir 2012; 28: 15015–15023. doi:10.1021/la302281n 22985189

[14] Celli BR, MacNee W, ATS/ERS Task Force Standards for the diagnosis and treatment of patients with COPD: a summary of the ATS/ERS position paper. Eur Respir J 2004; 23: 932–946. doi:10.1183/09031936.04.00014304 15219010

[15] Hankinson JL, Odencrantz JR, Fedan KB Spirometric reference values from a sample of the general U.S. population. Am J Respir Crit Care Med 1999; 159: 179–187. doi:10.1164/ajrccm.159.1.9712108 9872837

[16] Centers for Disease Control and Prevention National Center for Health Statistics *Third National Health and Nutrition Examination Survey III Spirometry Procedure Manual*. wwwn.cdc.gov/nchs/data/nhanes3/manuals/spiro.pdf. Date last accessed: November 8 2018.

[17] Pascoe S, Locantore N, Dransfield MT, et al. Blood eosinophil counts, exacerbations, and response to the addition of inhaled fluticasone furoate to vilanterol in patients with chronic obstructive pulmonary disease: a secondary analysis of data from two parallel randomised controlled trials. Lancet Respir Med 2015; 3: 435–442. doi:10.1016/S2213-2600(15)00106-X 25878028

[18] Watz H, Tetzlaff K, Wouters EFM, et al. Blood eosinophil count and exacerbations in severe chronic obstructive pulmonary disease after withdrawal of inhaled corticosteroids: a post-hoc analysis of the WISDOM trial. Lancet Respir Med 2016; 4: 390–398. doi:10.1016/S2213-2600(16)00100-4 27066739

[19] Bafadhel M, Peterson S, De Blas MA, et al. Predictors of exacerbation risk and response to budesonide in patients with chronic obstructive pulmonary disease: a post-hoc analysis of three randomised trials. Lancet Respir Med 2018; 6: 117–126. doi:10.1016/S2213-2600(18)30006-7 29331313

[20] Yun JH, Lamb A, Chase R, et al. Blood eosinophil count thresholds and exacerbations in patients with chronic obstructive pulmonary disease. J Allergy Clin Immunol 2018; 141: 2037–2047.e2010. doi:10.1016/j.jaci.2018.04.010 29709670 PMC5994197

[21] Siddiqui SH, Guasconi A, Vestbo J, et al. Blood eosinophils: a biomarker of response to extrafine beclomethasone/formoterol in chronic obstructive pulmonary disease. Am J Respir Crit Care Med 2015; 192: 523–525. doi:10.1164/rccm.201502-0235LE 26051430 PMC4595668

[22] Rabe KF, Martinez FJ, Ferguson GT, et al. A Phase III study of triple therapy with budesonide/glycopyrrolate/formoterol fumarate metered dose inhaler 320/18/9.6 μg and 160/18/9.6 μg using co-suspension delivery technology in moderate-to-very severe COPD: the ETHOS study protocol. Respir Med 2019; 158: 59–66. doi:10.1016/j.rmed.2019.08.010 31605923

[23] Suissa S Run-in bias in randomised trials: the case of COPD medications. Eur Respir J 2017; 49: 1700361. 28642311 10.1183/13993003.00361-2017

[24] Suissa S, Ariel A Triple therapy trials in COPD: a precision medicine opportunity. Eur Respir J 2018; 52: 1801848 10.1183/13993003.01848-2018. 30545959

[25] Ernst P, Gonzalez AV, Brassard P, et al. Inhaled corticosteroid use in chronic obstructive pulmonary disease and the risk of hospitalization for pneumonia. Am J Respir Crit Care Med 2007; 176: 162–166. doi:10.1164/rccm.200611-1630OC 17400730

[26] Calverley PMA, Anderson JA, Celli B, et al. Salmeterol and fluticasone propionate and survival in chronic obstructive pulmonary disease. N Engl J Med 2007; 356: 775–789. doi:10.1056/NEJMoa063070 17314337

[27] Suissa S, Patenaude V, Lapi F, et al. Inhaled corticosteroids in COPD and the risk of serious pneumonia. Thorax 2013; 68: 1029–1036. doi:10.1136/thoraxjnl-2012-202872 24130228 PMC3812880

[28] Janson C, Larsson K, Lisspers KH, et al. Pneumonia and pneumonia related mortality in patients with COPD treated with fixed combinations of inhaled corticosteroid and long acting β_2_ agonist: observational matched cohort study (PATHOS). BMJ 2013; 346: f3306. doi:10.1136/bmj.f3306 23719639 PMC3666306

[29] Janson C, Stratelis G, Miller-Larsson A, et al. Scientific rationale for the possible inhaled corticosteroid intraclass difference in the risk of pneumonia in COPD. Int J Chron Obstruct Pulmon Dis 2017; 12: 3055–3064. doi:10.2147/COPD.S143656 29089754 PMC5654780

[30] Hollis S, Jorup C, Lythgoe D, et al. Risk of pneumonia with budesonide-containing treatments in COPD: an individual patient-level pooled analysis of interventional studies. Int J Chron Obstruct Pulmon Dis 2017; 12: 1071–1084. doi:10.2147/COPD.S128358 28435240 PMC5389656

[31] Ferguson GT, Rabe KF, Martinez FJ, et al. Triple therapy with budesonide/glycopyrrolate/formoterol fumarate with co-suspension delivery technology versus dual therapies in chronic obstructive pulmonary disease (KRONOS): a double-blind, parallel-group, multicentre, phase 3 randomised controlled trial. Lancet Respir Med 2018; 6: 747–758. doi:10.1016/S2213-2600(18)30327-8 30232048

