# Supplementary material for: Efficacy and safety of two doses of budesonide/formoterol fumarate metered dose inhaler in COPD
Source: ERJ Open Res. 2020 Apr 27;6(2):00187-2019. doi: 10.1183/23120541.00187-2019 (PMC7184113; doi:10.1183/23120541.00187-2019)
Supplement: Supplementary file 1 [file Hanania_PT009003_SOPHOS_manuscript_RESUBMITTED_SUPPPL_INFO_23Mar20.docx]

Online data supplement

# Supplementary methods

## *Definition of COPD exacerbations and severity of exacerbations*

During this study, chronic obstructive pulmonary disease (COPD) exacerbations were defined as changes beyond normal day-to-day variation in a patient’s usual COPD symptoms that lasted for ≥2 days, were acute in onset, and may have warranted a change in regular medication. Changes must have included at least one major COPD symptom (dyspnoea, sputum volume and sputum colour) and at least one other major or minor COPD symptom (cough, wheeze, sore throat, cold symptoms [rhinorrhoea or nasal congestion] and fever without other cause). Investigators could still define an event as an exacerbation if symptoms were acute or had progressed rapidly and required treatment <2 days from onset, or if overall clinical findings supported the diagnosis of a COPD exacerbation, but the patient had not experienced a worsening of one or more major COPD symptom and at least one other major or minor symptom. In these cases, the investigator had to justify the decision for defining the event as an exacerbation and record it in the electronic case report form.

A COPD exacerbation was defined as moderate if it required the use of systemic corticosteroids and/or antibiotics for ≥3 days (a single depot injectable dose of corticosteroids was considered equivalent to a 3-day course of systemic corticosteroids) and as severe if it resulted in COPD-related hospitalisation or death. A COPD exacerbation that did not meet any requirements to be classified as moderate/severe was considered mild.

## *Key exclusion criteria*

Patients with COPD due to α_1_-antitrypsin deficiency, or other respiratory disorders such as lung cancer and cystic fibrosis, were excluded. Patients must have been able to demonstrate the correct metered dose inhaler administration technique.

## *Prohibited medications*

Prohibited COPD medications, including short-acting and long-acting bronchodilators (alone or as part of fixed-dose combination therapies), oral β-agonists and theophylline (at doses >400 mg/day) were discontinued at the start of screening. Foods and beverages containing xanthine or caffeine, and caffeine-containing medications, were prohibited for ≥6 hours before, and for the duration of, each in-clinic study visit.

## *Analysis populations*

The intent-to-treat (ITT) population, defined as all patients who were randomised to and received any amount of the study drug, was used for sensitivity analyses. Patients were analysed according to the treatment they were assigned at randomisation. Data obtained after discontinuation of treatment, but before withdrawal from the study, were included. The modified ITT population was a subset of the ITT population, defined as all patients with post-randomisation data obtained before treatment discontinuation (including patients who took less than one full dose of study drug). Any data collected after completion of, or discontinuation from, randomised study drug were excluded. Patients were analysed according to randomised treatment group. The safety population was defined as all patients who were randomised and received study drug (including patients who took less than one full dose).

## *Efficacy endpoints for Ex-US approach*

The primary and secondary endpoints, and the time frames for the analysis of each, differed depending on regional regulatory requirements. Two registration approaches, US and Ex-US, were used in the study. The main manuscript describes the analysis using the US approach. The endpoints and analysis for the Ex-US approach are described below. For the Ex-US approach, the primary endpoint was the change from baseline in morning pre-dose trough forced expiratory volume in 1 second (FEV_1_) over 24 weeks, analysed using the efficacy estimand. The same analysis using an attributable estimand was the first secondary endpoint.

Further secondary endpoints were: the percentage of patients achieving a minimal clinically important difference ≥4 units in St George’s Respiratory Questionnaire (SGRQ) total score (responder rate) over 24 weeks; change from baseline in average daily rescue medication use over 24 weeks; change from baseline in the Exacerbations of Chronic Pulmonary Disease Tool (EXACT) total score [1] over 24 weeks; Transition Dyspnoea Index (TDI) focal score [2] over 24 weeks; time to first moderate/severe COPD exacerbation; and time to first clinically important deterioration (CID). A CID was defined as either a ≥100 mL decrease from baseline in trough FEV_1_; ≥4 points increase from baseline in SGRQ total score; a TDI focal score of ≤−1 point; or treatment-emergent moderate-or-severe COPD exacerbation occurring up to week 52. The rate of moderate/severe exacerbations over the treatment period was also assessed.

Subgroup analyses of change from baseline in morning pre-dose trough FEV_1_ over 24 weeks were performed according to patients’ history of moderate/severe COPD exacerbations in the previous 12 months (1 or ≥2) and baseline blood eosinophil count (<150 or ≥150 cells/mm^3^).

## *Efficacy analyses*

The efficacy estimand was the primary estimand of interest for analysis of the primary and secondary endpoints for both the US and Ex-US registration approaches. The efficacy estimand was defined as the effect of the randomised treatment in all patients, assuming all patients continued treatment for the duration of the study, regardless of actual compliance. The attributable estimand, used for secondary analysis of the primary endpoint, was defined as the effect of treatment in patients attributable to the randomised treatment and considered treatment discontinuation due to lack of tolerability or efficacy as unfavourable outcomes.

For both the US and Ex-US registration approaches, the primary endpoint was analysed using a repeated measures linear mixed model. The model included treatment, visit, treatment-by-visit interaction and inhaled corticosteroid use at screening as categorical covariates, and baseline FEV_1_, blood eosinophil count and percent reversibility to albuterol sulfate as continuous covariates. Contrasts were used to obtain estimates of the treatment differences at week 12 and over 24 and 52 weeks, and each post-randomisation visit. Two-sided p-values and point estimates with two-sided 95% confidence intervals were produced for each treatment difference.

Analyses of rescue medication use (US and Ex-US approaches), EXACT total score (Ex-US approach), TDI focal score (Ex-US approach) and other related endpoints used similar repeated measures models to the primary endpoint, with their respective baseline measures as covariates. SGRQ responder analyses (US and Ex-US approaches) were completed using a logistic regression model. Time to CID (Ex-US approach) and time to first moderate/severe COPD exacerbation (US and Ex-US approaches) were analysed using Cox regression, and the rate of COPD exacerbations (US and Ex-US approaches) was analysed using negative binomial regression. The analysis models for CID and exacerbation endpoints included post-bronchodilator FEV_1_ % predicted, blood eosinophil count, country (exacerbations only), baseline COPD exacerbation history, ICS use at screening and treatment as covariates. The post-hoc analysis to estimate the rate of moderate/severe COPD exacerbations for each 4-week period was performed using repeated measures generalised estimating equation (GEE) negative binomial regression, adjusting for the same covariates as above with the addition of time interval and treatment by time interval interactions.

Each eosinophil subgroup was analysed separately using the same model as for the overall population analysis. To further evaluate the impact of baseline eosinophil counts on the change from baseline in morning pre-dose trough FEV_1_ at week 12 (primary endpoint) and the rate of moderate/severe COPD exacerbations, a series of locally weighted scatter plot smoothing (LOESS) curves were produced. For the FEV_1_ endpoint, the model used the change from baseline at week 12 and baseline eosinophil count from each patient to plot the change from baseline by treatment group across the range of observed eosinophil values in the study. For COPD exacerbations, pooled estimates of rates for ten-patient blocks based on sorted baseline eosinophil values were plotted against eosinophil values to avoid the influence of outliers.

For the US approach, the proposed sample size of 1860 patients (620 per treatment group) with 15% dropout provided approximately 90% power to detect a difference in the primary endpoint of 40 mL between budesonide/formoterol fumarate dihydrate metered dose inhaler (BFF MDI) and formoterol fumarate dihydrate metered dose inhaler (FF MDI) at week 12. For the Ex-US approach, the same sample size of 1860 patients, with 30% dropout, provided approximately 96% power to detect a difference in the primary endpoint of 40 mL between BFF MDI and FF MDI over 24 weeks.

Data processing, statistical screening, descriptive reporting and analysis of the efficacy and safety data was performed using SAS^®^ (Version 9.2 or higher) (Cary, NC, USA).

# Supplementary results

## *Efficacy for the Ex-US approach*

For the primary endpoint of change from baseline in morning pre-dose trough FEV_1_ over 24 weeks, analysed using the efficacy estimand, BFF MDI 320/10 µg and 160/10 µg demonstrated statistically significant improvements *versus* FF MDI (least squares mean differences 39 mL [p=0.0003] and 34 mL [p=0.0012], respectively (table E2). Analysis using the attributable estimand (secondary endpoint) provided similar results (table E2).

The time to first moderate/severe COPD exacerbation endpoint was the same in both the US and Ex-US approaches (table 2 and table E2). However, in the Ex-US approach, the treatment difference between BFF MDI 320/10 μg and FF MDI was not statistically significant due to the Type I error control for this approach (which included the TDI endpoint).

Both doses of BFF MDI demonstrated statistically significant improvements *versus* FF MDI for time to first CID, EXACT total score, rescue medication use and percentage of SGRQ responders over 24 weeks. Mean (standard deviation [SD]) baseline EXACT total scores were similar across treatment groups (BFF MDI 320/10 µg, 36.7 [12.1]; BFF MDI 160/10 µg, 36.3 [11.4]; FF MDI, 36.5 [11.8]). The mean (SD) Baseline Dyspnoea Index focal score was similar for all treatment groups (BFF MDI 320/10 µg, 6.0 [2.0]; BFF MDI 160/10 µg, 5.9 [1.9]; FF MDI, 6.0 [2.0]; table 1). Treatment differences in TDI focal score over 24 weeks did not reach statistical significance for either dose of BFF MDI *versus* FF MDI (table E2).

Similar to the US approach, subgroup analyses revealed that the improvements in morning pre-dose trough FEV_1_ over 24 weeks with BFF MDI compared with FF MDI were driven by patients with a baseline blood eosinophil count ≥150 cells/mm^3^ (figure E4). The number of COPD exacerbations in the previous 12 months (1 *versus* ≥2 exacerbations) appeared to not affect morning pre-dose trough FEV_1_ over 24 weeks (figure E5).

## *Deaths*

Of the 22 deaths in the study (on- and post-treatment), seven were due to cardiovascular causes (BFF MDI 320/10 µg, n=3; BFF MDI 160/10 µg, n=3; FF MDI, n=1), seven were due to respiratory causes (BFF MDI 320/10 µg, n=1; BFF MDI 160/10 µg, n=3; FF MDI, n=3), three were due to cancer (BFF MDI 320/10 µg, n=2; BFF MDI 160/10 µg, n=1) and five were due to other causes (accidents, infections or unknown; BFF MDI 160/10 µg, n=1; FF MDI, n=4). No deaths were considered drug-related.

## *Post-hoc analyses to assess acute ICS withdrawal effect*

Since acute ICS withdrawal has previously been associated with an increase in exacerbation rates, post-hoc analyses were performed to investigate the rate of moderate/severe exacerbations according to ICS use at baseline. The benefit of BFF MDI *versus* FF MDI on the rate of moderate/severe exacerbations was clearly demonstrated in both patients who were taking an ICS prior to study entry and patients who were not taking an ICS prior to study entry (table E3).

The annualised rate of exacerbations was estimated via GEE modelling for each 4-week period by treatment (figure E6). The analysis showed that there was some overlap for 3 months during the mid-portion of the exposure period. However, both doses of BFF MDI separated from FF MDI during the first 4 months and last 5 months of treatment, a pattern inconsistent with an acute ICS withdrawal effect.

# References

1. Jones PW, Chen WH, Wilcox TK, Sethi S, Leidy NK. Characterizing and quantifying the symptomatic features of COPD exacerbations. *Chest* 2011; 139: 1388-1394.

2. Witek TJ, Jr., Mahler DA. Minimal important difference of the transition dyspnoea index in a multinational clinical trial. *Eur Respir J* 2003; 21: 267-272.

TABLE E1 Percentage of patients with a first confirmed pneumonia event at weeks 12, 24 and 52 (safety population)

|  | BFF MDI  320/10 µg  (N=619) | BFF MDI  160/10 µg  (N=617) | FF MDI  10 µg  (N=607) |
| --- | --- | --- | --- |
| Patients with first confirmed pneumonia  event, % (95% CI) |  |  |  |
| Week 12 | 0.3 (0.1, 1.4) | 0.9 (0.4, 2.1) | 1.1 (0.5, 2.3) |
| Week 24 | 1.0 (0.4, 2.5) | 1.6 (0.8, 3.2) | 1.7 (0.9, 3.3) |
| Week 52 | 3.7 (1.8, 7.5) | 5.0 (2.6, 9.4) | 3.8 (2.2, 6.7) |

BFF: budesonide/formoterol fumarate dihydrate; CI: confidence interval; FF: formoterol fumarate dihydrate; MDI: metered dose inhaler.

TABLE E2 Primary, secondary and additional efficacy endpoints (Ex-US approach; modified intent-to-treat population; efficacy estimand unless stated otherwise)

|  | BFF MDI  320/10 μg  N=619 | BFF MDI 160/10 μg  N=617 | FF MDI  10 µg  N=607 |
| --- | --- | --- | --- |
| Primary endpoint |  |  |  |
| Change from baseline in morning pre-dose trough FEV_1_ (mL) over 24 weeks | | | |
| N  LSM (SE)  LSM difference *versus* FF MDI (95% CI)  p-value | 578  63 (7.9)  39 (18, 59)  0.0003 | 590  59 (7.7)  34 (14, 55)  0.0012 | 559  24 (8.0)  – –  – |
| Secondary endpoints |  |  |  |
| Change from baseline in morning pre-dose trough FEV_1_ (mL) over 24 weeks (attributable estimand) | | | |
| N  LSM (SE)  LSM difference *versus* FF MDI (95% CI)  p-value | 612  49 (8.2)  48 (27, 70)  <0.0001 | 612  44 (7.9)  44 (23, 65)  <0.0001 | 601  1 (8.3)  – –  – |
| Time to first moderate/severe COPD exacerbation | | | |
| Patients with exacerbation, n (%)  HR *versus* FF MDI  (95% CI)  p-value | 220 (35.5)  0.827 (0.688, 0.995)  0.0441^#^ | 223 (36.1)  0.803 (0.668, 0.966)  0.0198 | 241 (39.7)  – –  – |
| Time to first CID^¶^ | | | |
| Patients with ≥1 CID, n (%)  HR *versus* FF MDI (95% CI)  p-value | 469 (75.8)  0.830 (0.730, 0.944)  0.0044 | 462 (74.9)  0.783 (0.689, 0.890)  0.0002 | 491 (80.9)  – –  – |
| Change from baseline in average daily rescue medication use (puffs/day) over 24 weeks | | | |
| N  LSM (SE)  LSM difference *versus* FF MDI (95% CI)  p-value | 612  −1.0 (0.10)  −0.36 (−0.61, −0.11)  0.0052 | 610  −1.0 (0.09)  −0.34 (−0.59, −0.09)  0.0088 | 599  −0.6 (0.10) – –  – |
| Percentage of patients achieving an MCID of ≥4 units in SGRQ total score over 24 weeks | | | |
| Responders, n (%)  Difference *versus* FF MDI, % (95% CI)  p-value | 320 (52.81)  10.01 (4.130, 15.890)  0.0009 | 326 (53.18)  11.57 (5.710, 17.440)  0.0001 | 254 (42.62)  – –  – |
| Change from baseline in the EXACT total score over 24 weeks | | | |
| N  LSM (SE)  LSM difference *versus* FF MDI (95% CI)  p-value | 614  −2.6 (0.33)  −1.35 (−2.230, −0.460)  0.0029 | 613  −2.8 (0.33)  −1.53 (−2.420, −0.650)  0.0007 | 597  −1.2 (0.34)  – –  – |
| TDI focal score over 24 weeks | | | |
| N  LSM (SE)  LSM difference *versus* FF MDI (95% CI)  p-value | 566  1.32 (0.110)  0.25 (−0.030, 0.540)  0.0824 | 578  1.30 (0.107)  0.23 (−0.050, 0.520)  0.1130 | 550  1.06 (0.111) – –  – |
| Additional endpoints |  |  |  |
| Rate of moderate/severe exacerbations, per year^+^ | | | |
| Adjusted rate of exacerbations (SE)  Rate ratio *versus* FF MDI (95% CI)  p-value | 0.93 (0.073)  0.67 (0.54, 0.82)  0.0001^##^ | 0.98 (0.076)  0.71 (0.58, 0.87)  0.0010^##^ | 1.39 (0.106)  – –  – |
| Time to first moderate/severe COPD exacerbation in patients with a history of ≥2 moderate/severe COPD exacerbations in the last 12 months | | | |
| Patients with exacerbation, n (%)  HR *versus* FF MDI (95% CI)  p-value | 93 (39.4) 0.783 (0.590, 1.039)  0.0904 | 97 (40.2) 0.799 (0.603, 1.059)  0.1178 | 106 (45.7) – –  – |

BFF: budesonide/formoterol fumarate dihydrate; CI: confidence interval; CID: clinically important deterioration; COPD: chronic obstructive pulmonary disease; EXACT: Exacerbations of COPD Tool; FEV_1_: forced expiratory volume in 1 second; FF: formoterol fumarate dihydrate; HR: hazard ratio; LSM: least squares mean; MCID: minimal clinically important difference; MDI: metered dose inhaler; SE: standard error; SGRQ: St George’s Respiratory Questionnaire; TDI: Transition Dyspnoea Index.

^#^Nominally significant (*p*<0.05 but not statistically significant after Type I error control).

^¶^CID defined as either a ≥100 mL decrease from baseline in trough FEV_1_; ≥4 points increase from baseline in SGRQ total score; a TDI focal score of ≤−1 point; or treatment-emergent moderate-or-severe COPD exacerbation occurring up to week 52.

^+^Rate of exacerbations per year = total number of exacerbations / total years of exposure across all patients for the treatment.
^##^Nominally significant as not included in the Type I error control.

TABLE E3 Model-estimated annual rates of moderate/severe COPD exacerbations by ICS use at baseline (modified intent-to-treat population; efficacy estimand)

|  | BFF MDI  320/10 µg  (N=619) | BFF MDI  160/10 µg  (N=617) | FF MDI  10 µg  (N=607) |
| --- | --- | --- | --- |
| Patients using ICS at baseline |  |  |  |
| N  Rate, per year  *Rate ratio versus FF MDI (95% CI)*  *p-value* | 482  1.00  0.69 (0.55, 0.88)  0.003 | 459  1.03  0.72 (0.57, 0.92)  0.007 | 465  1.44  – – |
| Patients not using ICS at baseline |  |  |  |
| N  Rate, per year  *Rate ratio versus FF MDI (95% CI)*  *p-value* | 137  0.73  0.61 (0.38, 0.95)  0.030 | 158  0.86  0.71 (0.47, 1.08)  0.107 | 142  1.21  – – |

BFF: budesonide/formoterol fumarate dihydrate; CI: confidence interval; COPD, chronic obstructive pulmonary disease; FF: formoterol fumarate dihydrate; ICS, inhaled corticosteroid; MDI: metered dose inhaler.


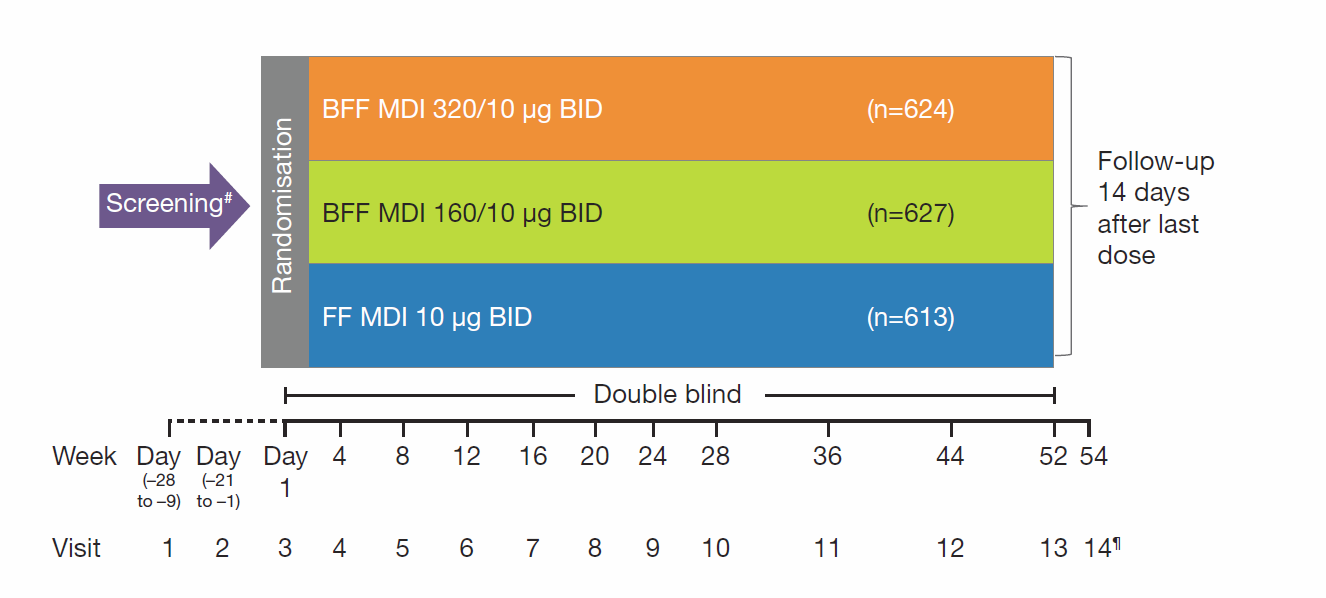


FIGURE E1 Study design.

^#^Screening period: 1–4 weeks (may be extended to 10 weeks if the patient experiences an exacerbation during screening).

^¶^Follow-up telephone call. For patients who discontinued study drug and did not complete at least one post-treatment data collection, a follow-up telephone call was required at least 14 days after the last study drug dose.

BID: twice daily; BFF: budesonide/formoterol fumarate dihydrate; FF: formoterol fumarate dihydrate; MDI: metered dose inhaler.


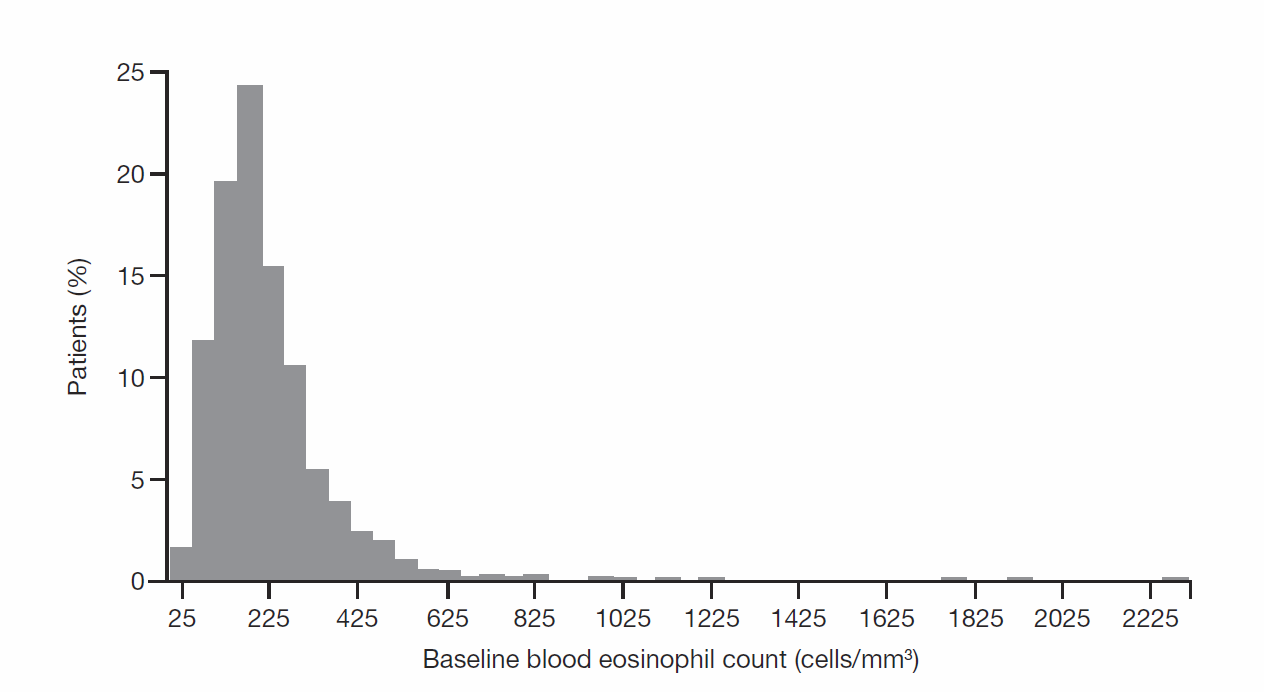


FIGURE E2 Baseline eosinophil distribution.


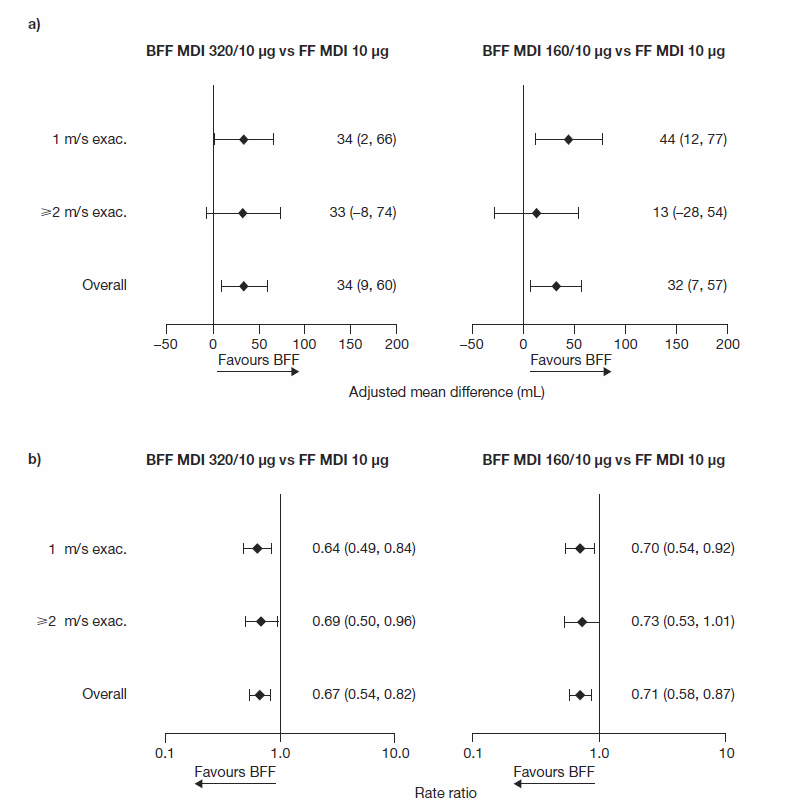


FIGURE E3 Change from baseline in morning pre-dose trough FEV_1_ at week 12 a) and rate of moderate/severe chronic obstructive pulmonary disease exacerbations b) by the history of moderate/severe exacerbations in the previous 12 months (modified intent-to-treat population; efficacy estimand).

BFF: budesonide/formoterol fumarate dihydrate; FEV_1_: forced expiratory volume in 1 second; FF: formoterol fumarate dihydrate; MDI: metered dose inhaler; m/s exac.: moderate/severe exacerbation.


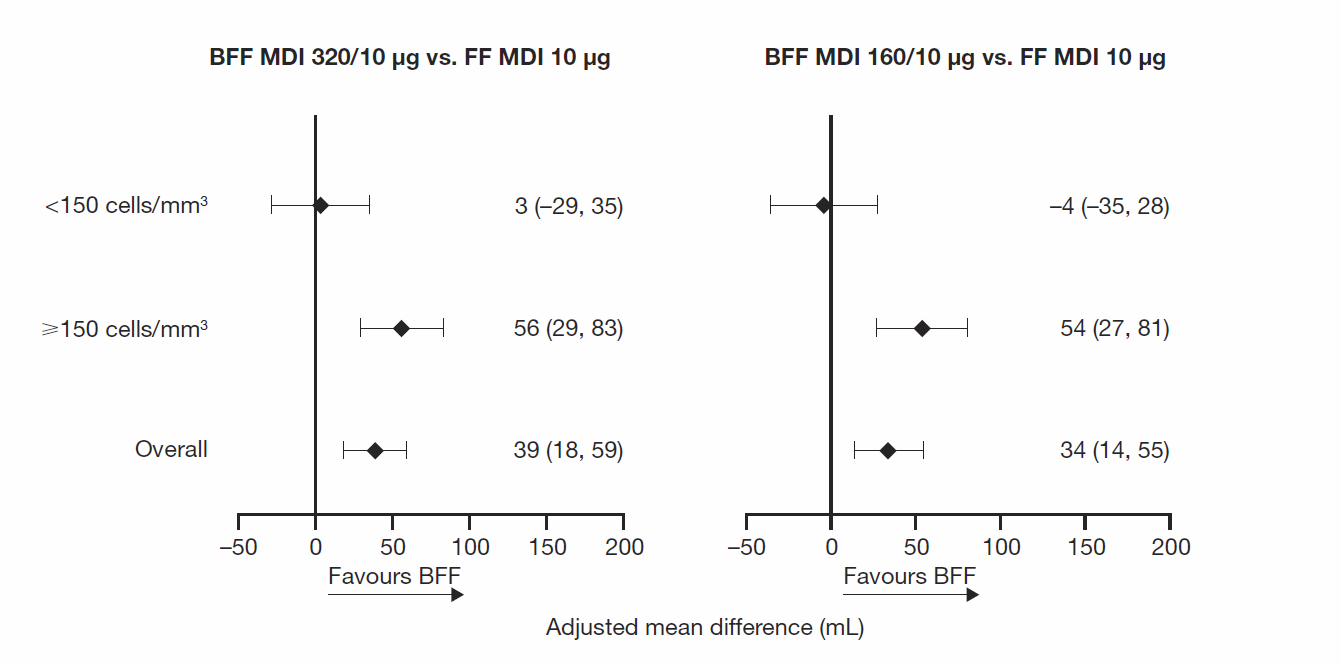


FIGURE E4 Change from baseline in morning pre-dose trough FEV_1_ over 24 weeks by eosinophil subgroups – Ex-US approach (modified intent-to-treat population; efficacy estimand).

BFF: budesonide/formoterol fumarate dihydrate; FF: formoterol fumarate dihydrate; MDI: metered dose inhaler.


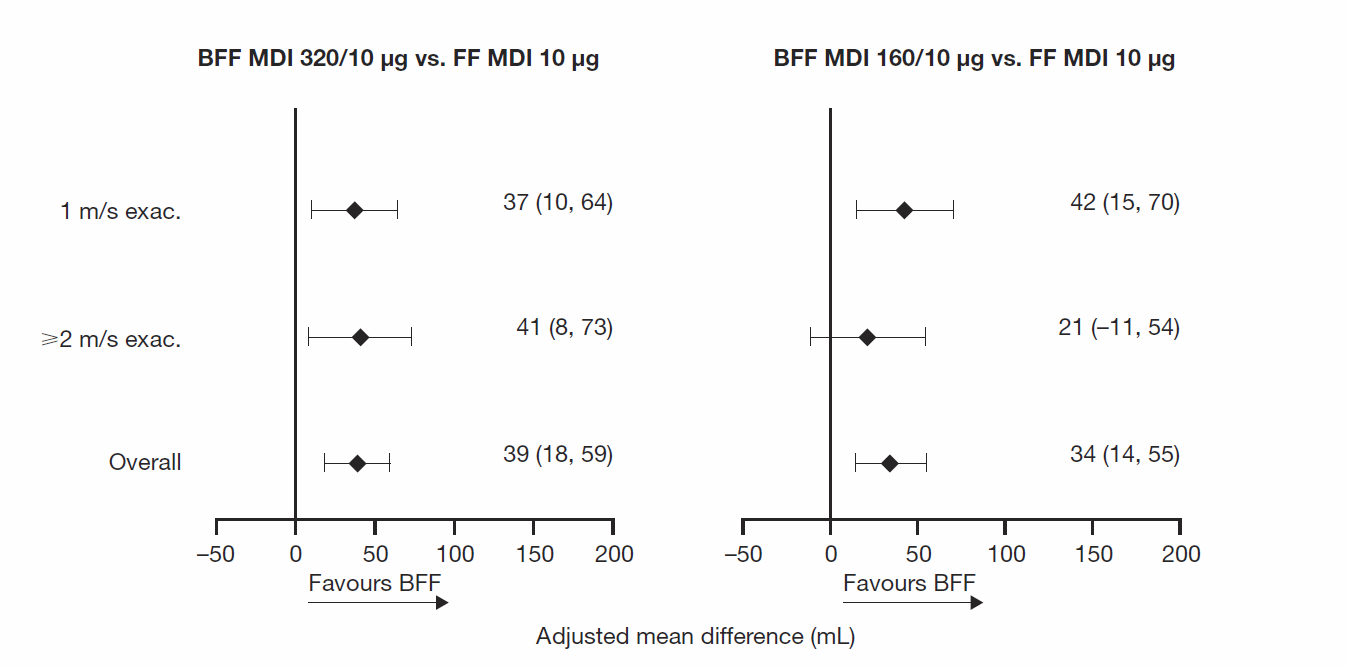


FIGURE E5 Change from baseline in morning pre-dose trough FEV_1_ over 24 weeks by the history of moderate/severe exacerbations in the previous 12 months – Ex-US approach (modified intent-to-treat population; efficacy estimand).

BFF: budesonide/formoterol fumarate dihydrate; FEV_1_: forced expiratory volume in 1 second; FF: formoterol fumarate dihydrate; MDI: metered dose inhaler; m/s exac: moderate/severe exacerbation.


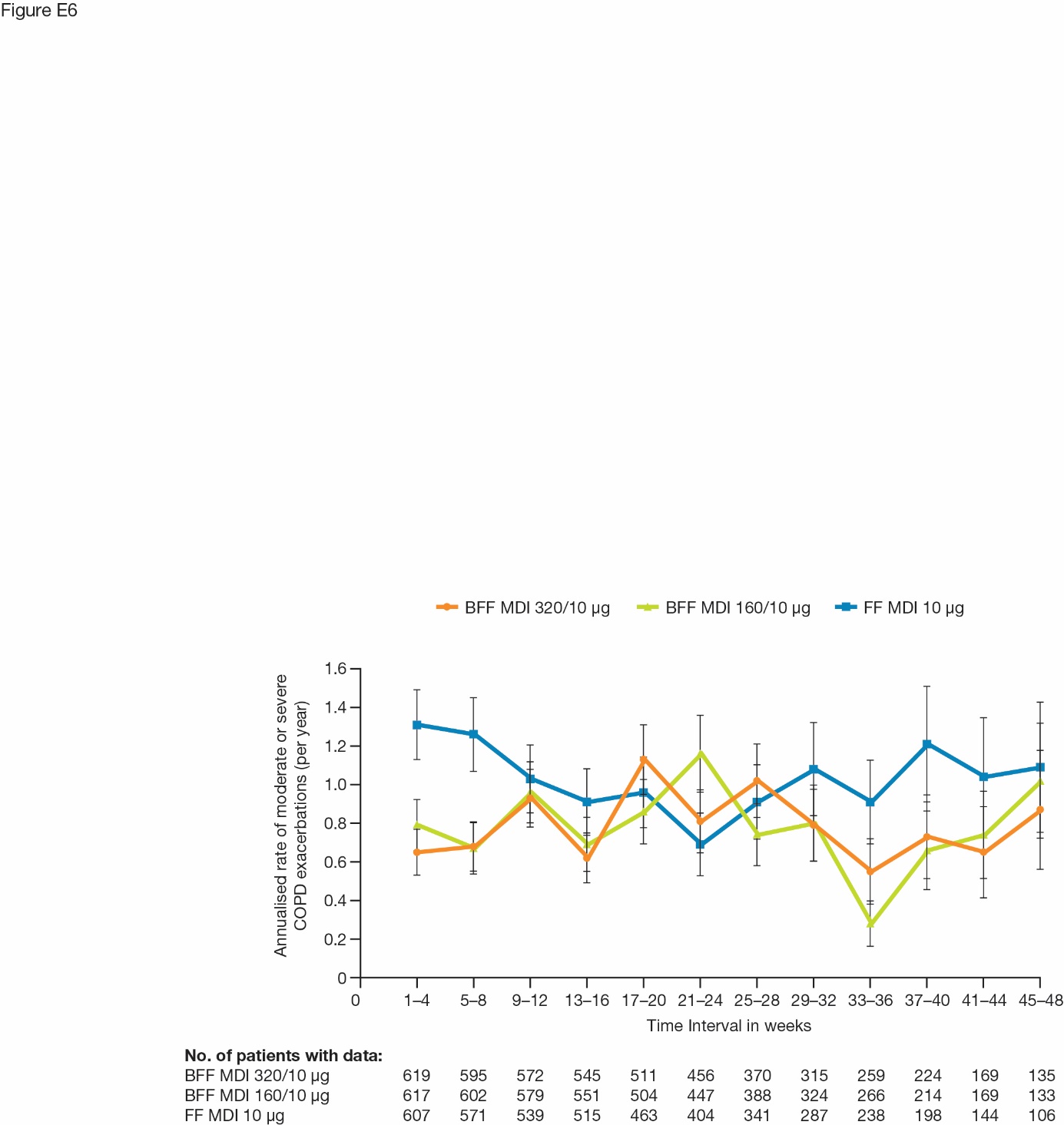


FIGURE E6 The annualized rate of moderate/severe COPD exacerbations by 4-week intervals (modified intent-to-treat population; GEE model).

BFF: budesonide/formoterol fumarate dihydrate; COPD: chronic obstructive pulmonary disease; FF: formoterol fumarate dihydrate; GEE: generalised estimating equation; MDI: metered dose inhaler.
